# 1844. Mortality associated with *Stenotrophomonas maltophilia* bloodstream infection in patients with leukemia

**DOI:** 10.1093/ofid/ofac492.1473

**Published:** 2022-12-15

**Authors:** Samuel L Aitken, Joslyn Rudoni, Kayleigh R Marx, Frank P Tverdek, Samuel A Shelburne, Pablo C Okhuysen

**Affiliations:** Michigan Medicine, Ann Arbor, Michigan; Cleveland Clinic, Cleveland, Ohio; The University of Texas MD Anderson Cancer Center, Houston, Texas; Seattle Cancer Care Alliance, Seattle, Washington; MD Anderson, Houston, Texas; The University of Texas MD Anderson Cancer Center, Houston, Texas

## Abstract

**Background:**

Patients with leukemia are disproportionately vulnerable to infections caused by *S. maltophilia* and mortality approaches 100% in patients with hemorrhagic pneumonia. Trimethoprim-sulfamethoxazole (SXT) is the drug of choice for treatment of infections caused by *S. maltophilia* however allergies, toxicity, and resistance may preclude its use. We sought to characterize clinical outcomes of patients with leukemia and *S. maltophilia* bloodstream infection (BSI).

**Methods:**

Patients with leukemia are disproportionately vulnerable to infections caused by *S. maltophilia* and mortality approaches 100% in patients with hemorrhagic pneumonia. Trimethoprim-sulfamethoxazole (SXT) is the drug of choice for treatment of infections caused by *S. maltophilia* however allergies, toxicity, and resistance may preclude its use. We sought to characterize clinical outcomes of patients with leukemia and *S. maltophilia* bloodstream infection (BSI).

**Results:**

Ninety-six patients with leukemia and *S. maltophilia* BSI were identified. The most common diagnosis was acute myeloid leukemia (60%); 31% had prior stem cell transplant. The most common single identified source was respiratory (31%) followed by central venous catheter (CVC, 26%). A single clear source was unable to be identified in 33%.Overall 14-day mortality was 31%. After excluding eleven patients who died within 24 hours, most patients received SXT (72%), followed by ceftazidime (40%), tigecycline (TGC, 40%), and minocycline (26%). SXT was associated with reduced mortality (hazard ratio [HR] 0.15, 95% confidence interval [CI] 0.05 – 0.44, p < 0.01) while TGC was associated with increased mortality (HR 3.13, 95% CI 1.00 – 9.73, p = 0.05). All patients who died were neutropenic at baseline and none had a CVC infection.

**Conclusion:**

*S. maltophilia* BSI is associated with high mortality in patients with leukemia, particularly those with neutropenia at baseline. CVC infection alone was not associated with mortality. SXT is associated with reduced mortality relative to other antimicrobials. Alternative agents should be used with caution in these vulnerable patients.

**Disclosures:**

**Samuel L. Aitken, PharmD, MPH**, Entasis Therapeutics: Advisor/Consultant|GlaxoSmithKline: Advisor/Consultant|Melinta: Grant/Research Support|Shionogi: Advisor/Consultant **Pablo C. Okhuysen, MD**, AstraZeneca: Stocks/Bonds|Beam Therapeutics: Stocks/Bonds|Biontech: Stocks/Bonds|Deinove: Grant/Research Support|Ferring: Advisor/Consultant|Glaxo Smith Kleine: Stocks/Bonds|Johnson and Johnson: Stocks/Bonds|Melinta: Grant/Research Support|Merck Sharp & Dohme Corp: Grant/Research Support|Moderna: Stocks/Bonds|Napo Pharmaceuticals: Advisor/Consultant|Napo Pharmaceuticals: Grant/Research Support|Novavax: Stocks/Bonds|Pfizer: Stocks/Bonds|Summit: Advisor/Consultant|Summit: Grant/Research Support.

